# Supplementary material for: Drug repurposing for aging research using model organisms
Source: Aging Cell. 2017 Jun 16;16(5):1006–15. doi: 10.1111/acel.12626 (PMC5595691; doi:10.1111/acel.12626)
Supplement: Supplementary file 7 — Data S1 Zip‐Archive of all report cards. [file ACEL-16-1006-s007.zip › RC_RDF.pdf]

RDF

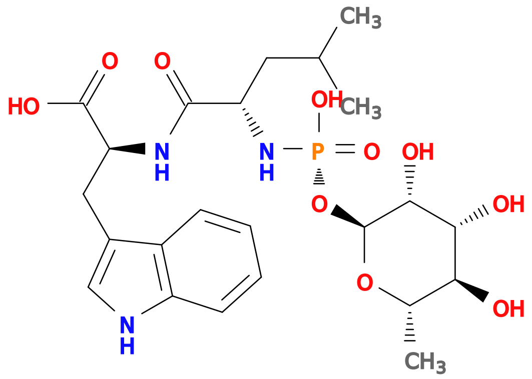

Database identifiers

ChEMBLCompound	CHEMBL479579
CHEBI	45353

Ranking

	Rank	Score
Drosophila	502/697	0.271
C. elegans	587/591	0.0

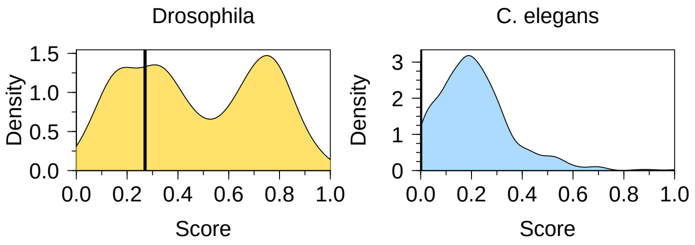

	Ageing implication	Domain conservation	Binding site conservation	Binding affinity	Bioavailability	Lipinski	Promiscuity	Purchasability	Drug approval	**Total**
Drosophila	0.792	0.752	0.898	0.875	(0.9)	-0.15	-0.0	0.0	0.0	0.271
C. elegans	0.792	0.869	0.896	0.875	0.2	-0.15	-0.0	0.0	0.0	0.0

Names

- **phosphoramidon**
- (2S)-2-(2S)-2-(S)-hydroxy(2S,3R,4R,5R,6S)-3,4,5-trihydroxy-6-methyltetrahydro-2H-pyran-2-yloxyphosphorylamino-4-methylpentanoylamino-3-(1H-indol-3-yl)propanoic acid (non-preferred name)
- N-(N-(((6-deoxy-alpha-L-mannopyranosyl)oxy)hydroxyphosphinyl)-L-leucyl)-L-tryptophan
- N-alpha-l-rhamnopyranosyloxy(hydroxyphosphinyl)-l-leucyl-l-tryptophan

Roles

bacterial metabolite, EC 3.4.24.11 (neprilysin) inhibitor, EC 3.4.24.71 (endothelin-converting enzyme 1) inhibitor

Status

Approved drug (according to ChEMBL)	No
Number of Rule of 5 violations	3
Binding affinity to original target in log units (RF-Score prediction)	6.95
Burns *C. elegans* bioavailability prediction	-14.67

Compound Target Characteristics

Neprilysin

Best gene implication in ageing for this target family came from gene P08473 annotated in UniProt release 2014_02. Annotation GO subterm of 7568 (aging) was Inferred from Expression Pattern

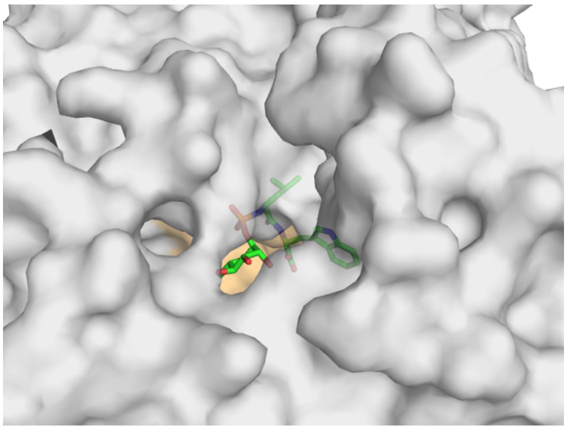

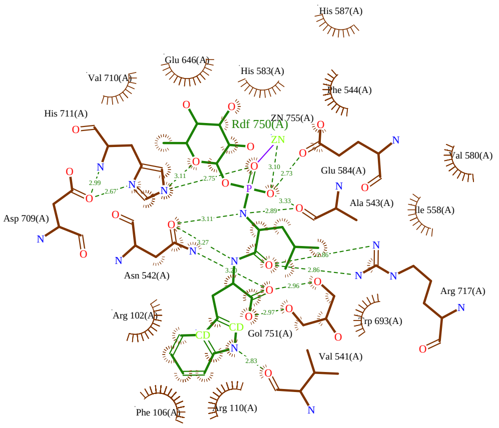

protein	amino acids contacts (binding site)																
PDB:1dmt:chainA:P08473	R	F	R	V	N	A	F	I	V	H	E	H	E	W	V	H	R
sp:P08473:NEP_HUMAN	R	F	R	V	N	A	F	I	V	H	E	H	E	W	V	H	R
sp:P07861:NEP_RAT	R	F	R	V	N	A	F	I	V	H	E	H	E	W	V	H	R
sp:Q61391:NEP_MOUSE	R	F	R	V	N	A	F	I	V	H	E	H	E	W	V	H	R
tr:Q9XZ01:Q9XZ01_DROME	S	F	S	V	N	A	F	I	V	H	E	H	E	W	V	H	R
tr:Q9W436:Q9W436_DROME	S	F	S	V	N	A	F	I	V	H	E	H	E	W	V	H	R
tr:Q9I7I4:Q9I7I4_DROME	G	F	R	V	N	A	Y	I	V	H	E	H	E	W	I	H	R
tr:Q8T062:Q8T062_DROME	G	F	R	V	N	A	Y	I	V	H	E	H	E	W	I	H	R
sp:Q18673:NEPL1_CAEEL	S	F	R	V	N	A	F	I	V	H	E	H	E	W	P	H	R
sp:O16796:NPL11_CAEEL	G	F	R	V	N	A	F	I	V	H	E	H	E	W	E	H	R

	whole protein		domain-based		contact-based	
protein	ident	simil	ident	simil	ident	simil
PDB:1dmt:chainA:P08473	1.0	1.0	1.0	1.0	1.0	1.0
sp:P08473:NEP_HUMAN	1.0	1.0	1.0	1.0	1.0	1.0
sp:P07861:NEP_RAT	0.94	0.99	0.95	0.99	1.0	1.0
sp:Q61391:NEP_MOUSE	0.94	0.99	0.95	0.99	1.0	1.0
tr:Q9XZ01:Q9XZ01_DROME	0.32	0.71	0.38	0.78	0.88	0.87
tr:Q9W436:Q9W436_DROME	0.34	0.67	0.44	0.81	0.88	0.87
tr:Q9I7I4:Q9I7I4_DROME	0.27	0.56	0.35	0.64	0.82	0.9
tr:Q8T062:Q8T062_DROME	0.26	0.54	0.35	0.64	0.82	0.9
sp:Q18673:NEPL1_CAEEL	0.3	0.7	0.37	0.76	0.88	0.9
sp:O16796:NPL11_CAEEL	0.29	0.67	0.38	0.79	0.88	0.86

Nep4 (FBgn0038818) associated phenotypes

locomotor behavior defective

(Information from FlyBase)

nep-1 (UniProt:Q18673) annotation

Function: Probable cell surface protease. Required to control the neuronal innervation of pharyn-

geal pumping. (PubMed:16081104).
Cofactor: Zn(2+)Note=Binds 1 zinc ion per subunit. ;
Subcellular location: Membrane; Single-pass type II membrane protein.
Tissue specificity: Specifically expressed in pharyngeal cells and a single head neuron. (PubMed:16081104).

(Information from UniProt)

nep-2 (UniProt:O16796) annotation
Function: Probable cell surface protease.
Cofactor: Zn(2+)Note=Binds 1 zinc ion per subunit. ;
Subcellular location: Cell membrane ; Single-pass type II membrane protein

(Information from UniProt)
